# Angiogenesis PET Tracer Uptake (^68^Ga-NODAGA-E[(cRGDyK)]_2_) in Induced Myocardial Infarction in Minipigs

**DOI:** 10.3390/diagnostics6020026

**Published:** 2016-06-17

**Authors:** Thomas Rasmussen, Bjarke Follin, Jens Kastrup, Malene Brandt-Larsen, Jacob Madsen, Thomas Emil Christensen, Karsten Pharao Hammelev, Philip Hasbak, Andreas Kjær

**Affiliations:** 1 Department of Clinical Physiology, Nuclear Medicine & PET and Cluster for Molecular Imaging, Rigshospitalet and University of Copenhagen, Blegdamsvej 9, 2100 Copenhagen, Denmark; malene.brandt-larsen@regionh.dk (M.B.-L.); jacob.madsen@regionh.dk (J.M.); thomas.emil.christensen@regionh.dk (T.E.C.); philip.hasbak@regionh.dk (P.H.); andreas.kjaer@regionh.dk (A.K.); 2 Cardiology Stem Cell Centre, Department of Cardiology, Rigshospitalet, University of Copenhagen, Blegdamsvej 9, 2100 Copenhagen, Denmark; bjarke.follin.larsen@regionh.dk (B.F.); jens.kastrup@regionh.dk (J.K.); 3 Department of Experimental Medicine, University of Copenhagen, Blegdamsvej 3B, 2100 Copenhagen, Denmark; kah@sund.ku.dk

**Keywords:** positron-emission-tomography, angiogenesis, myocardial infarction

## Abstract

Angiogenesis is part of the healing process following an ischemic injury and is vital for the post-ischemic repair of the myocardium. Therefore, it is of particular interest to be able to noninvasively monitor angiogenesis. This might, not only permit risk stratification of patients following myocardial infarction, but could also facilitate development and improvement of new therapies directed towards stimulation of the angiogenic response. During angiogenesis endothelial cells must adhere to one another to form new microvessels. α_v_β_3_ integrin has been found to be highly expressed in activated endothelial cells and has been identified as a critical modulator of angiogenesis. ^68^Ga-NODAGA-E[c(RGDyK)]_2_ (RGD) has recently been developed by us as an angiogenesis positron-emission-tomography (PET) ligand targeted towards α_v_β_3_ integrin. In the present study, we induced myocardial infarction in Göttingen minipigs. Successful infarction was documented by ^82^Rubidium-dipyridamole stress PET and computed tomography. RGD uptake was demonstrated in the infarcted myocardium one week and one month after induction of infarction by RGD-PET. In conclusion, we demonstrated angiogenesis by noninvasive imaging using RGD-PET in minipigs hearts, which resemble human hearts. The perspectives are very intriguing and might permit the evaluation of new treatment strategies targeted towards increasing the angiogenetic response, e.g., stem-cell treatment.

**Figure 1:**
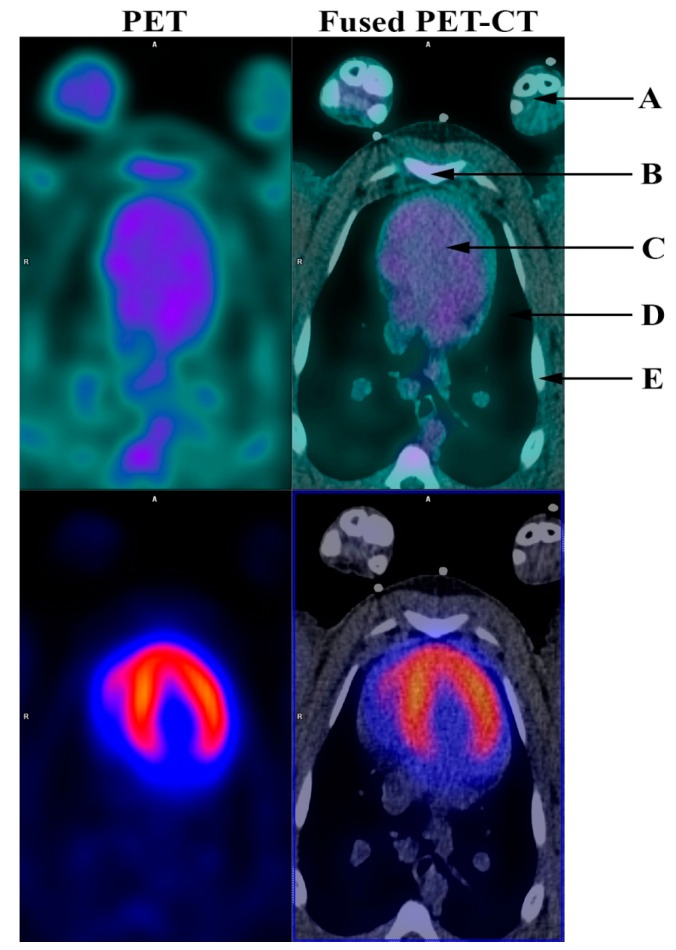
Angiogenesis PET (RGD PET) (top row) and ^82^Rb dipyridamole stress PET (bottom row) before induced myocardial infarction. (**A**) Front limbs; (**B**) Sternum; (**C**) Heart; (**D**) Lungs; (**E**) Ribs. Angiogenesis is part of the healing process following an ischemic injury and is vital for the post-ischemic repair of the myocardium. It is associated with the remodeling of the left ventricle and thus prognosis following myocardial infarction [1]. Therefore, it is of particular interest to be able to noninvasively monitor angiogenesis. This might not only permit risk stratification of patients following myocardial infarction, but could also facilitate development and improvement of new therapies directed towards stimulation of the angiogenic response. During angiogenesis endothelial cells must adhere to one another to form new microvessels. This is a process modulated by the extracellular matrix including integrins. Specifically, α_v_β_3_ integrin is highly expressed in activated endothelial cells and has been identified as a critical modulator of angiogenesis and is therefore a potential target for directly imaging angiogenesis [2,3]. Existing noninvasive imaging methods directed towards the evaluation of angiogenesis have however been somewhat limited, possibly due to the fact that myocardial angiogenesis following myocardial infarction might be focal and therefore difficult to detect. Furthermore, most of the previous studies in angiogenesis imaging have been performed in smaller animals, mostly rats [4,5,6,7,8,9,10,11,12,13]. ^68^Ga-NODAGA-E[c(RGDyK)]_2_ (RGD) has recently been developed by us as an angiogenesis positron-emission-tomography (PET) ligand targeted towards α_v_β_3_ integrin [14]. In the present study, we induced myocardial infarction in Göttingen minipigs [15]. Successful infarction was documented by ^82^Rubidium (^82^Rb)-dipyridamole stress PET and computed tomography (CT) (Siemens mCT, Siemens, 128-slice CT, Knoxville, USA). RGD uptake was demonstrated in the infarcted myocardium one week and one month after induction of infarction by RGD-PET. The study was approved by the National Authority in Denmark (approval number: 2014-15-0201-00191). During the PET acquisition minipigs were anesthetized as described in detail previously [15]. Baseline ^82^Rb rest and stress myocardial perfusion were performed the week prior to induction of myocardial infarction as a 7 min dynamic PET myocardial perfusion rest scan under administration of 1000–1200 MBq ^82^Rb followed by a 7 min dynamic dipyridamole stress PET-CT. Dipyridamole (140 µg/kg/min) was given as a continuous intravenous infusion over 4 min prior to ^82^Rb-tracer injection 3–5 min after the completion of dipyridamole infusion. The RGD-PET was performed as a 10 min ECG-gated scan 45 min after administration of 100 MBq RGD. PET images were analyzed using Cedars-Sinai Cardiac Suite (Cedars-Sinai Medical Center, Los Angeles, CA, USA) for Syngo. Via (Siemens, Knoxville, TN, USA). The figure shows RGD and ^82^Rb stress PET images before induction of myocardial infarction. ^82^Rb stress PET showed even distribution of ^82^Rb in the left ventricle while the RGD PET showed no RGD uptake.

**Figure 2:**
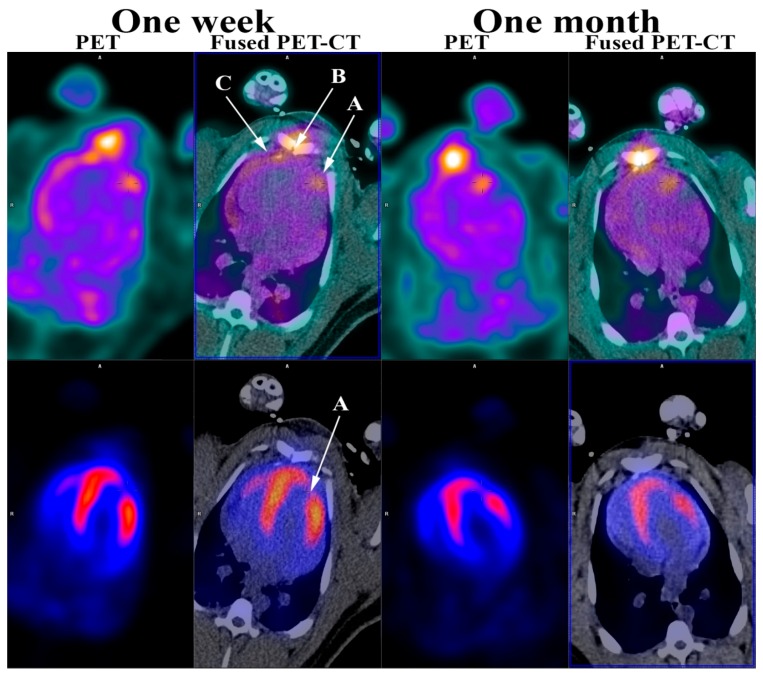
RGD (top row) and ^82^Rb stress PET (bottom row) one week and one month after induced myocardial infarction. (**A**) Myocardial infarction; (**B**) Sternotomy; (**C**) Pericardium. As shown, the ^82^Rb stress PET (bottom row) showed a myocardial perfusion defect in the anterior wall of the left ventricle myocardium one week and one month after induced myocardial infarction confirming a myocardial infarction corresponding to an area supplied by the ligated branch from LAD. This myocardial perfusion defect was also present at rest (not shown). Furthermore, the RGD PET (top row) showed RGD uptake in the infarcted myocardium one week and one month following myocardial infarction. In addition, RGD PET showed RGD uptake in the sternum after sternotomy and pericardium, most likely due to the opening as part of the infarct induction procedure. As previously mentioned, most of the previous work in angiogenesis imaging have been done in smaller animals. The minipig heart and the human heart are very much alike, which makes the findings in this study even more encouraging and adds to the few, mostly very small, studies performed in human [16,17,18]. The perspectives are very intriguing and might permit the evaluation of new treatment strategies targeted towards increasing the angiogenetic response, e.g., stem-cell treatment.
